# AKT inhibition is associated with chemosensitisation in the pancreatic cancer cell line MIA-PaCa-2

**DOI:** 10.1038/sj.bjc.6601037

**Published:** 2003-07-15

**Authors:** B N Fahy, M Schlieman, S Virudachalam, R J Bold

**Affiliations:** 1 Department of Surgical Oncology, University of California Davis Cancer Center, Sacramento, CA, USA

**Keywords:** AKT, BCL-2, NF-*κ*B, pancreas cancer

## Abstract

Activation of the serine/threonine kinase AKT is common in pancreatic cancer; inhibition of which sensitises cells to the apoptotic effect of chemotherapy. Of the various downstream targets of AKT, we examined activation of the NF-*κ*B transcription factor and subsequent transcriptional regulation of BCL-2 gene family in pancreatic cancer cells. Inhibition of either phosphatidylinositol-3 kinase or AKT led to a decreased protein level of the antiapoptotic gene BCL-2 and an increased protein level of the proapoptotic gene BAX. Furthermore, inhibition of AKT decreased the function of NF-*κ*B, which is capable of transcriptional regulation of the BCL-2 gene. Inhibiting this pathway had little effect on the basal level of apoptosis in pancreatic cancer cells, but increased the apoptotic effect of chemotherapy. The antiapoptotic effect of AKT activation in pancreatic cancer cells may involve transcriptional induction of a profile of BCL-2 proteins that confer resistance to apoptosis; alteration of this balance allows sensitisation to the apoptotic effect of chemotherapy.

Pancreatic cancer is a highly lethal malignancy resistant to the apoptosis-inducing effects of radio- and chemotherapy (Heinemann, 2002). Recent reports have demonstrated that the phosphatidylinositol-3 kinase (PI3K)/AKT pathway is a potent survival signal that may mediate resistance to the apoptotic effects of chemotherapy and radiation therapy in a variety of cancer types (Kandel and Hay, 1999; Brazil and Hemmings, 2001; Cantrell, 2001). Recent reports have suggested that AKT, a target of PI3K, is phosphorylated and thus activated under basal conditions in a variety of pancreatic cancer cell lines (Xue *et al*, 2000; Bondar *et al*, 2002; Samatar *et al*, 2002). The mechanism of AKT activation in pancreatic cancer remains unknown, although the majority of cell lines examined to date harbour constitutively activated AKT. Inhibition of PI3K, the upstream activator of AKT, has been shown to sensitise various pancreatic cancer cell lines to the apoptotic effect of chemotherapy *in vitro* (Ng *et al*, 2000; Perugini *et al*, 2000; Yao *et al*, 2002). Furthermore, using these cell lines in animal studies, inhibition of PI3K was well tolerated and increased the efficacy of chemotherapy *in vivo* (Ng *et al*, 2000; Bondar *et al*, 2002).

The downstream targets of AKT include regulators of apoptosis (BAD, caspase-9), glucose metabolism (glycogen kinase), various transcription factors (CREB and the forkhead family of transcription factors), and finally the I-kappaB kinases (IKKs). It remains unclear, however, which of these targets is involved in the antiapoptotic effect of AKT activation. The NF-*κ*B transcription factor appears to be involved in the apoptotic resistance of pancreatic cancer. This is based on observations of constitutive activation of NF-*κ*B in pancreatic cancer cells (Wang W *et al*, 1999) as well as the fact that inhibition of NF-*κ*B decreases cell survival (Shah *et al*, 2001) and enhances the apoptotic effect of chemotherapy in pancreatic cancer cells (Arlt *et al*, 2001).

NF-*κ*B transcription factors can regulate the expression of over 100 different genes dependent on the various functional forms of NF-*κ*B, the stimulus of NF-*κ*B activation and cell type examined. NF-*κ*B has been shown to regulate transcriptionally the expression of several members of the BCL-2 gene family, including BCL-X_L_ (Chen *et al*, 2000) and A1 (Wang CY *et al*, 1999). BCL-2 remains the prototypic antiapoptotic protein and the data are less clear as to whether NF-*κ*B regulates its transcription. There are reports of activation or inhibition as well as reports demonstrating no NF-*κ*B-dependent transcriptional effect in the BCL-2 promoter (Sohur *et al*, 1999; Catz and Johnson, 2001; Potoka *et al*, 2002). In pancreatic cancer, BCL-2 is frequently overexpressed and its overexpression confers chemo- and radioresistance and enhances tumorigenic and metastatic capability (Bold *et al*, 1999a,1999b; Nio *et al*, 2001; Su *et al*, 2001). However, it remains uncertain as to whether AKT or NF-*κ*B is involved in the mechanism of BCL-2 transcriptional activation in pancreatic cancer.

Therefore, given the preliminary cellular studies that demonstrate that the inhibition of PI3K/AKT sensitises pancreatic cancer to the apoptotic effect of chemotherapy, we sought to determine the molecular events that may mediate this effect. The current study examines the hypothesis that a survival signal from AKT activation is mediated by NF-*κ*B and subsequent transcriptional regulation of BCL-2 gene family members; furthermore, inhibition of this pathway sensitises pancreatic cancer cells to the apoptotic effect of gemcitabine.

## MATERIALS AND EXPERIMENTAL PROCEDURES

### Materials

All chemical reagents were purchased from Sigma Chemical Company (St Louis, MO, USA) unless otherwise specified. LY294002 was obtained from New England BioLabs (Beverly, MA, USA) and is a well-established inhibitor of PI3K-mediated activation of AKT (Cuenda and Alessi, 2000). Cell culture supplies and media were purchased from Becton Dickinson (San Diego, CA, USA) and Gibco/BRL Life Technologies (Gaithersburg, MD, USA), respectively. Plasmids for transfection experiments were purified using Qiagen's maxi kit (Valencia, CA, USA). The antibodies used included monoclonal antibodies to BCL-2, BAX, BAK, BCL-X_L_ and MCL-1 (BD Pharmingen, San Diego, CA, USA), a polyclonal antibody to phospho-AKT (serine-473) (Biosource International Inc., Camarillo, CA, USA), polyclonal antibody to AKT (Cell Signalling Technology, Inc., Beverly, MA, USA), a polyclonal antibody to actin (Santa Cruz Biotechnology, Inc., Santa Cruz, CA, USA) and a monoclonal antibody to the p50 subunit of NF-*κ*B (Oncogene Research Products, Boston, MA, USA). The luciferase assay kit was purchased from Promega Corp. (Madison, WI, USA). The dominant-negative PI3K and AKT constructs were kindly provided by Dr Hsing-Jien Kung (UC Davis). These constructs have been developed to inhibit specifically the enzymatic activity of PI3K and AKT and are used as molecular inhibitors of these signalling cascade intermediaries (Aoki *et al*, 1998; Osada *et al*, 1999).

### Cell culture

The human pancreatic adenocarcinoma cell lines MIA-PaCa-2 and PANC-1 were obtained from the American Type Culture Collection (Rockville, MD, USA). The MIA-PaCa-2 cell line has been previously demonstrated to harbour constitutively activated AKT (Samatar *et al*, 2002). Cells were cultured in Dulbecco's modified Eagle's medium supplemented with 10% fetal calf serum, sodium pyruvate, nonessential amino acids, L-glutamine and penicillin/streptomycin antibiotics. The cells were maintained in a humidified incubator containing 10% CO_2_ at 37°C. The cells underwent serum starvation for the 24 h prior to treatment with LY294002 or transient transfection.

### Western blotting

Following various treatments, cells were harvested by trypsinisation (trypsin 0.25% w v^−1^, 1 mM ethylenediaminetetraacetic acid). The cells were lysed in a lysis buffer containing 150 mM NaCl, 1% Triton X-100 and 25 mM Tris (pH 7.5). Debris was sedimented by centrifugation for 5 min at 12 000 **g**, and the supernatants were solubilised for 5 min at 100°C in Laemmli's sodium dodecyl sulphate–polyacrylamide gel electrophoresis (SDS–PAGE) sample buffer containing 100 mM dithiothreitol. Protein concentrations of the lysates were determined with a protein quantitation kit (Bio-Rad Laboratories, Hercules, CA, USA), and 50 *μ*g of each sample was separated on a 10% SDS–PAGE gel. Separated polypeptides were then electrophoretically transferred to 0.2-mm nitrocellulose membranes (Schleicher & Schuell, Keene, NH, USA). Membranes were blocked for 1 h in a Tris-buffered saline-Tween (25 mM Tris, pH 8.0, 150 mM NaCl, and 0.05% Tween-20) containing 5% (w v^−1^) nonfat dried milk. The blots were then probed overnight with primary antibodies and developed using species-specific secondary and tertiary antisera. Immunoreactive material was detected by the enhanced chemiluminescence technique (Amersham).

### BCL-2 and NF-*κ*B promoter activity

Transcriptional regulation of the BCL-2 gene was determined using a BCL-2 promoter/luciferase construct. A 3.7 kb fragment of the 5′ untranslated region just upstream of the ATG initiation codon of BCL-2 (Miyashita *et al*, 1994) was generously provided by Dr Toshiyuki Miyashita (National Children's Medical Research Center, Tokyo, Japan). This fragment of the BCL-2 promoter was cloned into the pGL3-basic luciferase reporter plasmid (Promega Corp.). The NF-*κ*B luciferase plasmid contains four tandem copies of the NF-*κ*B consensus sequence fused to a TATA-like promoter (Clontech, Palo Alto, CA, USA). BCL-2 promoter activity was normalised to the activity of the control Renilla luciferase reporter, pRL-TK (Promega Corp.). Following 6 h of pretreatment with the various inhibitors, the promoter/luciferase reporter plasmids, BCL-2 or NF-*κ*B (2.5 *μ*g), plus the control promoter/reporter, pRL-TK (0.25 *μ*g) were transiently transfected in MIA-PaCa-2 cells using lipofectin reagent (Life Technologies, Inc.). Fresh medium was applied after the cells had been in the transfection reagent for 8–12 h. The cells were harvested 24–48 h after transfection by gentle scraping and resuspended in luciferase lysis buffer.

Dual luciferase assays were performed according to the manufacturer's protocol (Promega Corp.). A volume of 20 *μ*l of sample supernatant was mixed with 100 *μ*l Luciferase Assay Reagent II followed by a 10 s reading of firefly luciferase activity using an Analytical Luminescence Laboratory Luminometer (Monolight 2010) followed by the addition of 100 *μ*l of Stop and Glo reagent™ (Promega Corp.). Dual luciferase assays were expressed as the ratio of firefly/Renilla RLUs. All experiments were performed in triplicate and repeated on at least three separate occasions; representative data from a single experiment are shown.

#### Site-directed mutagenesis of putative NF-*κ*B sites within the BCL-2 promoter

Putative NF-*κ*B sites within the BCL-2 promoter were identified using transcriptional element site software (Schug and Overton, 1997). A site that is homologous to the NF-*κ*B consensus sequence (5′GGGTNNYYCC3′) was altered by site-directed mutagenesis using a commercially available kit (Promega Corp.). A mutagenic oligonucleotide was synthesised to mutate two bases in the putative NF-*κ*B sequence at −736 nt (wild type: 5′GGGATTCCTGC3′; mutant: 5′GTTATTCCTGC3′). It has been recently shown that mutation of the second guanine base in an NF-*κ*B binding site essentially eliminates both p50/p50 and p50/p65 NF-*κ*B binding (Udalova *et al*, 2002). DNA sequencing was used to confirm the site-directed mutagenesis.

#### Electrophoretic mobility shift assay

Cell extracts were prepared using a commercially available nuclear extraction kit according to the manufacturer's protocol (Pierce, Rockford, IL, USA). Electrophoretic mobility shift assay (EMSA) was performed according to the provided protocol (Promega Corp.). In brief, a 21-mer oligonucleotide corresponding to a putative NF-*κ*B site in the BCL-2 promoter was radiolabelled with [*γ*-^32^P]ATP by a T4 kinase reaction. Nucleotides were purified by chromatography through a G-25 spin column (Roche Diagnostics Corp., Indianapolis, IN, USA) equilibrated in TE buffer. In all, 10 *μ*g of nuclear protein extract were incubated with the radiolabelled NF-*κ*B oligonucleotide for 20 min at room temperature. Supershift experiments were performed by incubating the radiolabelled NF-*κ*B oligonucleotide with 1 *μ*l of p50 antibody for 1 h on ice prior to incubation with the nuclear protein extracts. DNA : protein complexes were separated by electrophoresis through a nondenaturing 4% polyacrylamide gel in 0.5 × TBE at 100 V for 2.5 h. Autoradiographic films were developed following 18 h exposure to the gels (−20°C).

#### Determination of apoptotic cells by FACS analysis

Cell cycle analysis and quantification of apoptosis analysis was carried out as described previously using propidium iodide (PI) staining and fluorescence-activated cell sorting (FACS) (Bold *et al*, 1999a,1999b). In brief, following treatment, the cells were collected by gentle trypsinisation, washed in phosphate-buffered saline (PBS), and pelleted by centrifugation. The cells were fixed in 70% ethanol, washed twice in PBS and resuspended in PBS containing RNAse A (20 *μ*g ml^−1^). The cells were stained with PI (final concentration 0.1 mg ml^−1^) for 10 min at room temperature. The samples underwent FACS analysis (FL-3 channel) using a Beckman Coulter Counter Epics XL flow cytometer (Beckman Coulter, Inc., Miami, FL, USA). For each sample, 10 000 events were collected and stored for subsequent analysis using EXPO software (version 2.0; Applied Cytometry Systems, South Yorkshire, UK). Data were elaborated using Autofit feature of the Multicycle for Windows software (version 3.0, University of Washington, WA, USA) and expressed as fraction of cells in the different cycle phases. The percentage of cells in the sub-G_0_ phase was quantitated as an estimate of cells undergoing apoptosis.

## RESULTS

**Figure 1:**
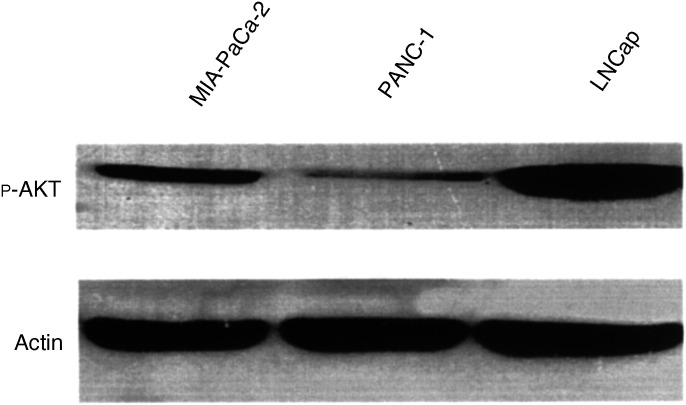
Levels of phospho-AKT (serine-473) and actin in the two human pancreatic cancer cell lines MIA-PaCa-2 and PANC-1, with the human prostate cancer cell line LNCaP as a positive control, which harbours a mutation in PTEN and thus demonstrates constitutive activation of PI3K (Davies *et al*, 1999).

**Figure 2:**
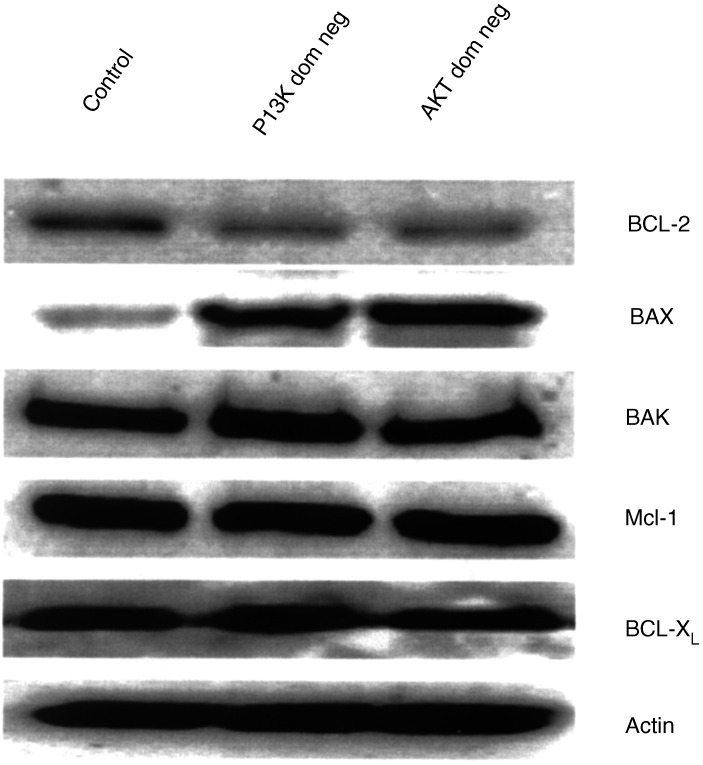
Effect of inhibition of PI3K or AKT (through transient transfection of the dominant-negative mutants (‘dom neg’) of these kinases) on BCL-2 family protein expression. Actin is shown for equivalency of loading.

We confirmed that the MIA-PaCa-2 cell line demonstrates the activation of AKT under basal conditions by Western blotting with a phospho-specific (serine 473) antibody (Figure 1

). The level of AKT activation in MIA-PaCa-2 is well below that of the human prostate cancer cell line LNCaP, which harbours a PTEN mutation and thus has high levels of PI3K activity (Davies *et al*, 1999). In comparison to MIA-PaCa-2, the pancreatic cancer cell line PANC-1 has very low levels of basal activation of AKT (Figure 1). The effect of PI3K/AKT inhibition on the levels of the BCL-2 gene family was determined by transiently transfecting MIA-PaCa-2 cells with dominant-negative mutants of either PI3K or AKT and performing Western blots. Both PI3K and AKT inhibition decreased BCL-2 expression and increased BAX expression without effect on MCL-1, BCL-X_L_ or BAK (Figure 2

).

**Figure 3:**
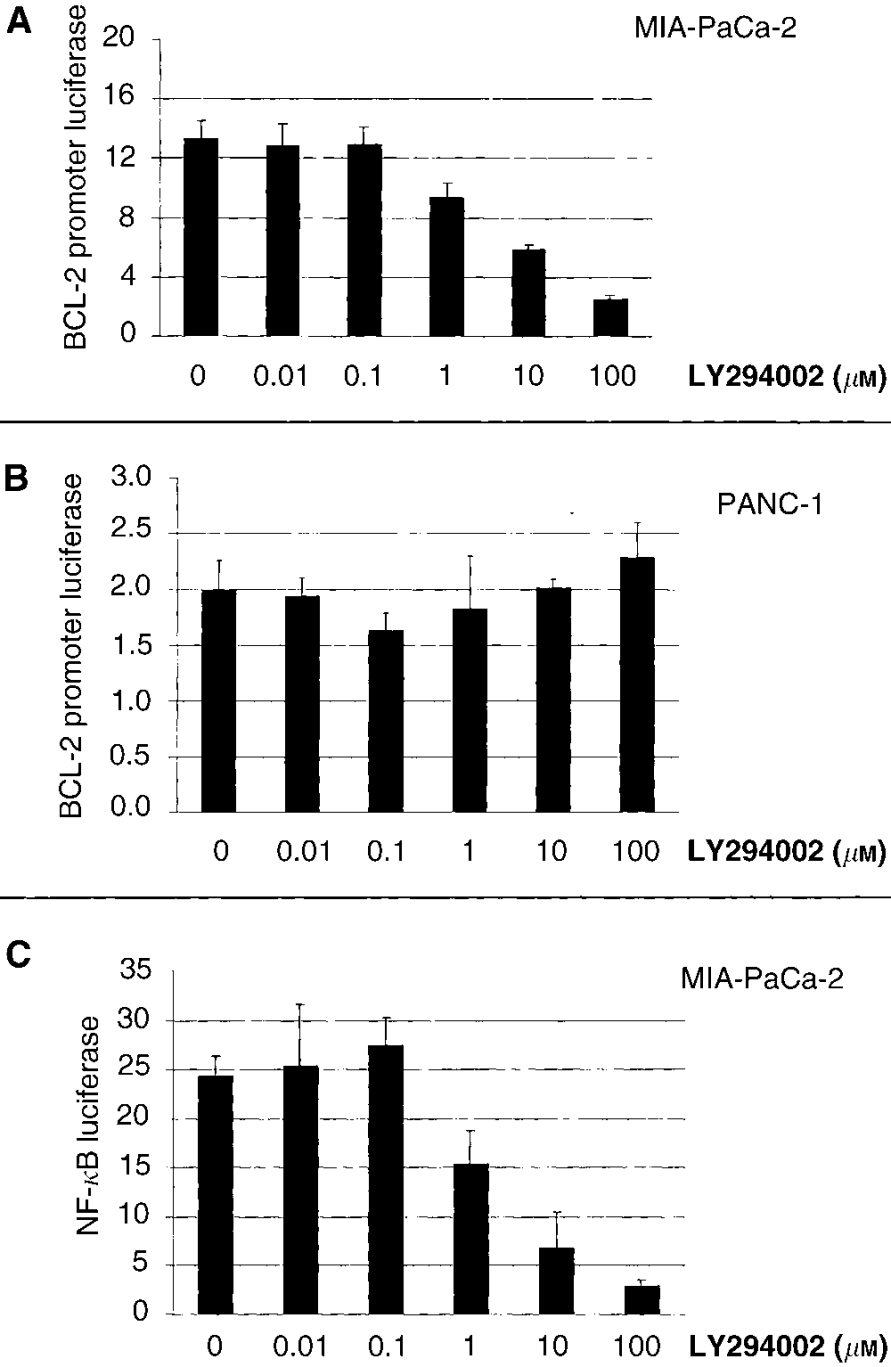
Treatment with the PI3K inhibitor, LY294002 inhibited the activation BCL-2 promoter in the MIA-PaCa-2 cell line in a dose-dependent manner(**A**) but without effect in the PANC-1 cell line (**B**). Furthermore, LY294002 inhibited NF-*κ*B transcriptional activity in MIA-PaCa-2 cells (**C**). Total lysate firefly luciferase was normalised to Renilla luciferase following dual transfection.

To determine whether these changes were mediated by alterations in gene transcription, we focused our investigation on the BCL-2 gene. To eliminate any potential effect of sequential transfections, we utilised the established pharmacologic inhibitor of PI3K, LY294002. Following treatment with LY294002, a dose-dependent reduction in BCL-2 promoter activity was observed only in the MIA-PaCa-2 cell line, but not in the PANC-1 cell line (Figure 3

), which has a much lower level of basal AKT activation. We examined similarly the transactivational function of NF-*κ*B, given its potential regulatory function of BCL-2 gene transcription. A parallel reduction in NF-*κ*B function was also observed following PI3K inhibition with LY294002 in the MIA-PaCa-2 cell line (Figure 3C).

**Figure 4:**
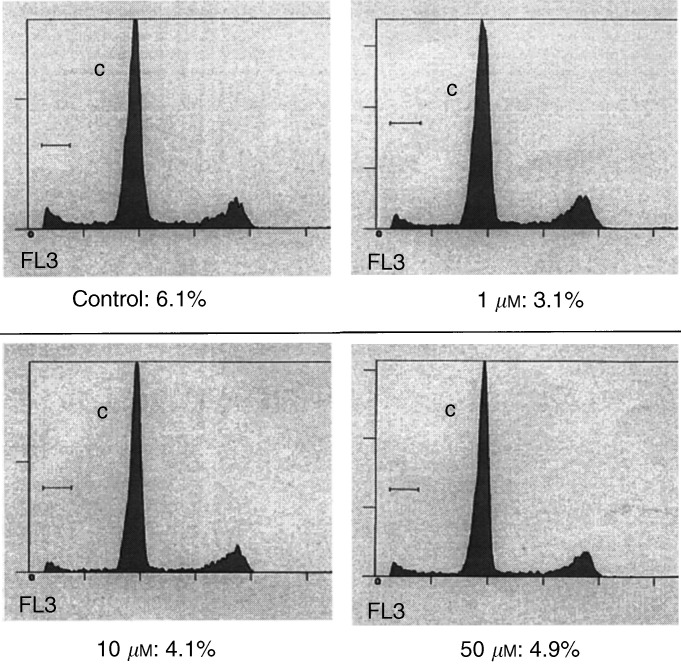
FACS analysis following 24 h of treatment with the indicated doses of the PI3K inhibitor, LY294002. Also shown is the relative percentage of cells in the sub-G_0_ phase of cell distribution, which corresponds to the fraction undergoing apoptosis. Data is graphed by number of events on x-axis and logarithmic value of flourescent intensity on y-axis.

This decrease in the content of an antiapoptotic protein (BCL-2) with an increase in the level of a proapoptotic protein (BAX) is a pattern consistent with a lower threshold for the induction of apoptosis. We determined whether these cells would undergo spontaneous apoptosis following PI3K inhibition alone. Using flow cytometry, we observed little change in the induction of apoptosis following treatment with a wide range of doses of LY294002 (Figure 4

). These cells exhibit a low level of spontaneous apoptosis, which was unchanged following treatment with any dose of LY294002. Of note, these doses were sufficient to decrease dramatically BCL-2 transcription as seen above, as well as protein levels (data not shown).

**Figure 5:**
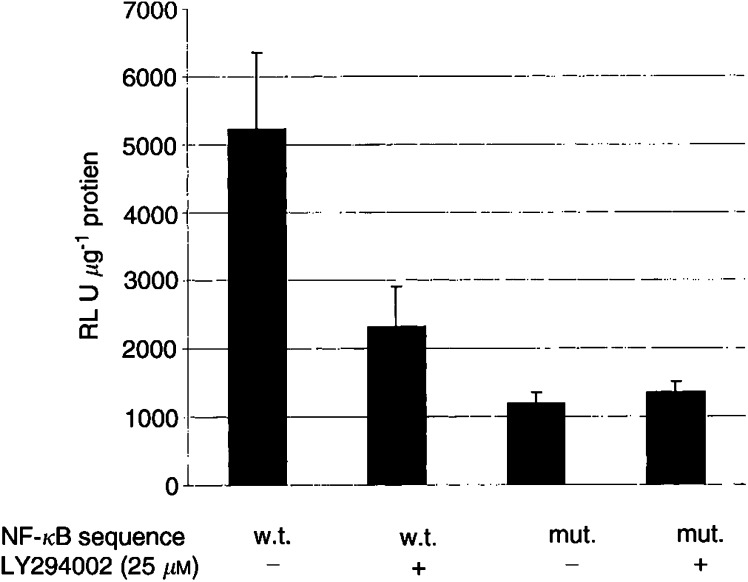
Effect of mutation of the NF-*κ*B site on BCL-2 promoter activity was examined using a full-length BCL-2 promoter/luciferase reporter containing a site-specific mutation (‘mut.’) compared to the wild-type BCL-2 promoter (‘w.t.’) in the absence or presence of LY294002. Total lysate RLUs were normalised for protein concentration of the cellular lysate for equivalency of analysis.

Having shown that the inhibition of AKT activation by PI3K reduces NF-*κ*B activity, BCL-2 promoter activity and subsequent protein expression, we next sought to more definitively establish the link between NF-*κ*B and BCL-2. To do this, a putative NF-*κ*B site within the BCL-2 promoter underwent site-directed mutagenesis in which two bases were mutated. BCL-2 promoter activity reduced by 77% following site-directed mutation of the putative NF-*κ*B site (Figure 5

). The effect of mutating this NF-*κ*B site in conjunction with PI3K inhibition on BCL-2 promoter activity was also examined. No further decrease in BCL-2 promoter activity was seen when PI3K inhibition was combined with mutation of the NF-*κ*B site (Figure 5). These data indicate that NF-*κ*B is the dominant transcription factor activated by PI3K in the regulation of BCL-2 promoter function, and that the site at −736 nt is the major NF-*κ*B site.

**Figure 6:**
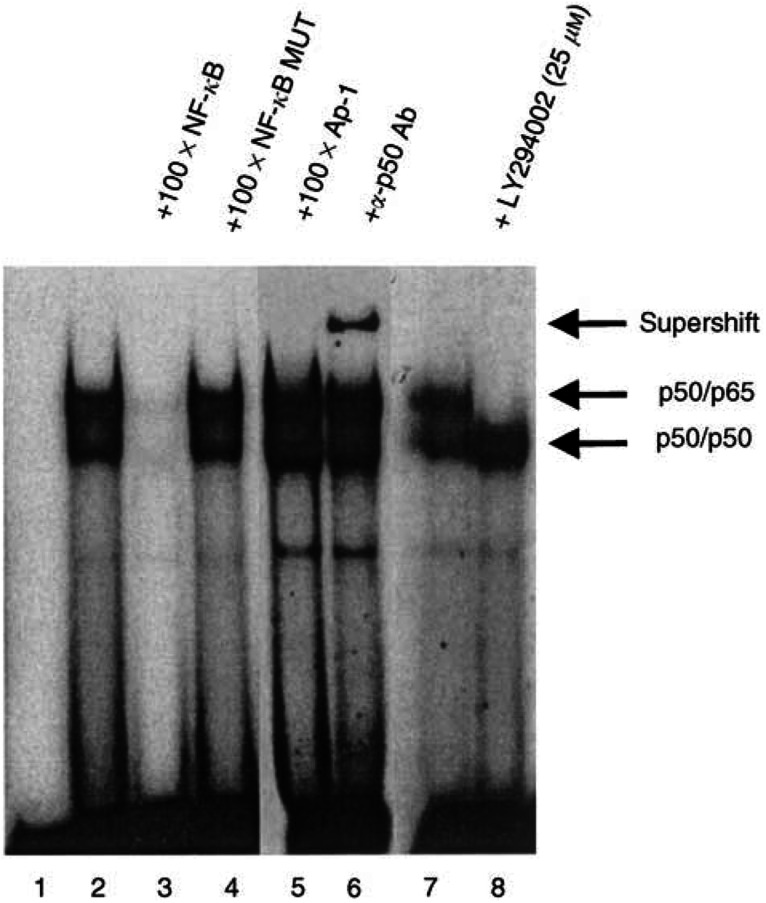
Examination of the NF-*κ*B site at −736 nt within the BCL-2 promoter by EMSA. MIA-PaCa-2 nuclear lysate was incubated with radiolabelled oligonucleotides corresponding to the NF-*κ*B site (except lane 1) and underwent cold competition (100 × molar excess) of nucleotides corresponding to the NF-*κ*B site (lane 3), the site-directed mutant of the NF-*κ*B site (lane 4), or an unrelated transcriptional site, AP-1 (lane 5). Supershift of the NF-*κ*B complex was achieved using an antibody to the p50 subunit of NF-*κ*B (lane 6). Treatment of the cells with LY294002 eliminated the upper p50/p65 band of the typical NF-*κ*B gelshift pattern (lane 8).

Final confirmation of the role of NF-*κ*B as mediator of the effect of PI3K on BCL-2 was achieved by EMSA. Using a radiolabelled 18-base oligonucleotide corresponding to the putative NF-*κ*B site in the BCL-2 promoter (−736 nt), the typical pattern of p50/p65 and p50/50 bands are observed (Figure 6

, lane 2). These bands were competed away by an excess of unlabelled NF-*κ*B oligonucleotide (lane 3), but not by either oligonucleotides representing the mutant NF-*κ*B sequence from the site-directed mutagenesis (lane 4) or an AP-1 consensus oligonucleotide (lane 5). The identity of the transcriptional element was confirmed by adding the antibody to the p50 subunit of NF-*κ*B to demonstrate a supershifted complex (lane 6). Finally, the effect of PI3K inhibition on this NF-*κ*B binding to the −736 nt site in the BCL-2 promoter was examined by treating the cells with LY294004 for 5 h prior to nuclear protein harvest. Of specific interest is the elimination of the upper p50/p65 complex only, with accentuation of the lower p50/p50 complex. This suggests that the NF-*κ*B signalling mediated by PI3K is primarily through p65 activity with little effect on p50 activity.

**Figure 7:**
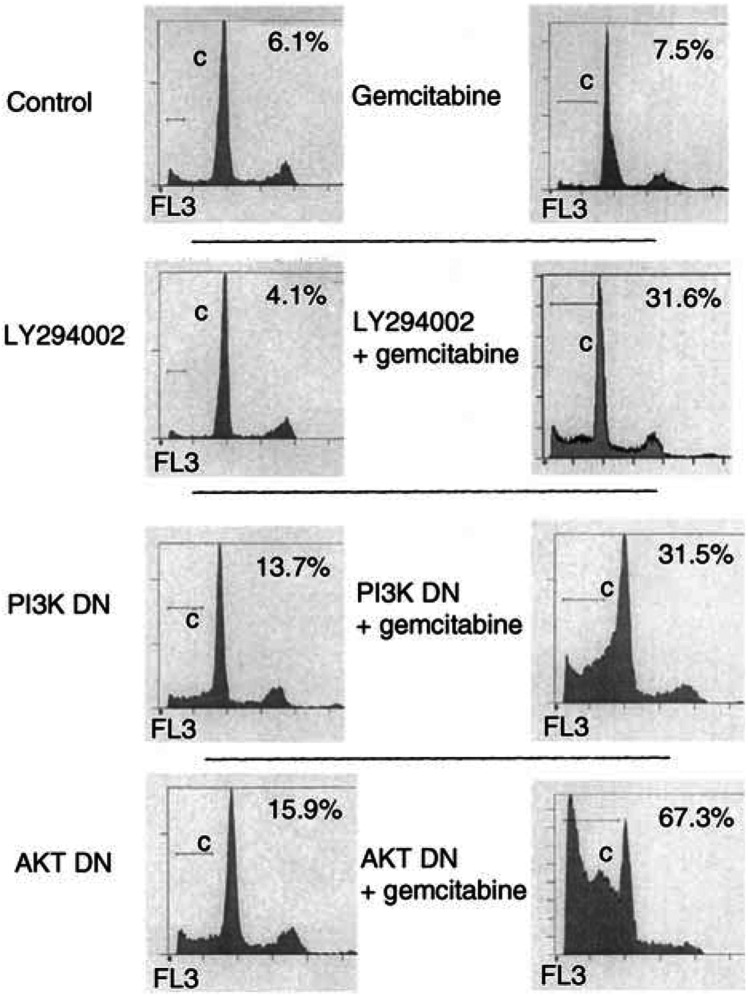
FACS analysis following indicated treatments of LY294002 (10 *μ*M), transient transfection with dominant-negative (DN) mutant forms of either PI3K or AKT alone or in combination with gemcitabine (100 *μ*M). Also shown is the relative percentage of cells in the sub-G_0_ phase of cell distribution, which corresponds to the fraction of cells undergoing apoptosis. Data is displayed in similar format to that of Figure 4.

As we observed very little apoptosis associated with the various treatments employed to inhibit PI3K or AKT, we subsequently investigated the cellular response to the inhibition of this signalling pathway as well as to exposure to gemcitabine, a standard chemotherapy for pancreatic cancer. Using FACS analysis, we observed a small increase in the induction of apoptosis with gemcitabine (Figure 7

), consistent with the literature that report little apoptotic effect of gemcitabine in these cells following short-term treatment (Xu *et al*, 2001). PI3K or AKT inhibition using the dominant-negative constructs induced apoptosis to a minor degree (Figure 7). However, there was a significant increase when the cells were treated with gemcitabine in the presence of PI3K or AKT inhibition (Figure 7). Therefore, the addition of PI3K or AKT inhibition converts gemcitabine into a potent apoptotic agent.

## DISCUSSION

Inhibition of the PI3K/AKT pathway has repeatedly and consistently been shown to sensitise pancreatic cancer cells *in vitro* and *in vivo* to the apoptotic effect of chemotherapy. The mechanism by which AKT activation in these cancer cells confers chemoresistance is unclear. AKT has been shown to have multiple targets, including mediators of apoptosis (e.g. caspase-9 and BAD), transcription factors (e.g. FKHD and CREB) and assorted kinases (e.g. raf, GSK-3 and IKK). There has been extensive investigation coupling AKT to IKK and subsequent NF-*κ*B activation, which can be a potent survival signal through transcription of various target genes. However, which genes confer this effect is undergoing further investigation.

The effect of modifying single members of the BCL-2 gene family on apoptotic response has been examined in a variety of cancer types and models. However, it is now accepted that while the levels of a single member of the BCL-2 gene family can be associated with apoptotic response, the overall balance of proapoptotic to antiapoptotic proteins (such as the BCL-2 : BAX ratio) sets the threshold for the induction of apoptosis in response to an external stimulus (Mirjolet *et al*, 2000). This is likely related to the function of these proteins through their ability to homo- and heterodimerise. Homodimers of proapoptotic proteins are sufficient for the induction of apoptosis; high levels of expression of antiapoptotic BCL-2 family members saturate the proapoptotic members and prevent the initiation of the apoptotic cascade (Mikhailov *et al*, 2001). Cancer cells are chemoresistant by virtue of an elevated threshold for the induction of apoptosis (termed ‘apopstat’). Indeed, dysregulation of BCL-2 gene family expression is common in most cancers, although the mechanisms are unclear.

Unlike other genetic events associated with cancer, altered expression of BCL-2 family members appears to occur at the level of transcription. A review of the reported effect of PI3K inhibition on BCL-2 family transcription reveals diverse functions that are dependent on cell type. In the JYTF-1 haematopoietic progenitor cell line, PI3K inhibition decreased MCL-1 levels, but had no effect on BCL-2 or BAX levels (Huang *et al*, 2000). On the other hand, in FDCP-1 (B-cell progenitor line) or PC12 (rat pheochromocytoma) cells, PI3K inhibition decreased BCL-2 gene transcription (Minshall *et al*., 1999; Pugazhenthi *et al*, 2000). Finally, in hippocampal neurons, PI3K inhibition decreased BCL-2 and increased BAX (Matsuzaki *et al*, 1999). Our data show that PI3K/AKT inhibition in these pancreatic cancer cells results in a decrease in BCL-2 and an increase in BAX without effect on other members of the BCL-2 family; furthermore, this effect appears limited to those cell lines that demonstrate AKT activation under basal conditions.

The specific mediators of BCL-2 transcription have been an active area of research. Pugazhenthi reported that the PI3K-mediated transcriptional regulation of BCL-2 was through transactivation of the CREB transcription factor (Pugazhenthi *et al*, 2000), although among the AKT-activated transcription factors, NF-*κ*B has been more consistently integrated into antiapoptotic signalling through regulation of either BCL-X_L_ or A1/Bfl-1 (Wang W *et al*, 1999). Whether NF-*κ*B functions as a transcriptional activator or repressor of BCL-2 remains unclear. Using the FL5.12 B-cell progenitor line, NF-*κ*B activation repressed transcription of the BCL-2 gene (Sohur *et al*. 1999). Conversely, in a model of androgen withdrawal from prostate cancer cells, NF-*κ*B activation induced BCL-2 gene transcription (Catz and Johnson, 2001). Heckman failed to identify a function NF-*κ*B site in the BCL-2 promoter of lymphoma cells harbouring a t(14:18) translocation (Heckman *et al*, 2002), although the functional NF-*κ*B site we have identified is within the promoter analysed by these investigators. It is clear from these various reports, that the integrity and function of PI3K/NF-*κ*B/BCL-2 signalling pathway is dependent on a variety of factors, including cell type, genetic background and transformed status. Of interest, we observed that PI3K inhibition did not further decrease BCL-2 promoter activity harbouring a mutated NF-*κ*B site, suggesting that NF-*κ*B alone was the major transcription factor activated by PI3K, and it functioned primarily at the −736 nt location.

Previous studies have demonstrated that the inhibition of PI3K sensitises pancreatic cancer to the apoptotic effect of chemotherapy; our studies provide data to define a signalling pathway that involves alterations in relative levels of BCL-2 family members. Inhibition of PI3K/AKT has minimal effect on the basal level of apoptosis, although it leads to a lower threshold for the induction of apoptosis in response to chemotherapy. These data suggest that targeted inhibition of the PI3K/AKT pathway may not be an appropriate therapy when administered alone, but may improve the efficacy of standard chemotherapeutic agents. An improved understanding of the mechanism(s) by which pancreatic cancer cells evade apoptotic signals may result in the development of novel therapies that can restore apoptotic sensitivity in this highly lethal malignancy.
